# Empagliflozin protects mice against diet-induced obesity, insulin resistance and hepatic steatosis

**DOI:** 10.1007/s00125-022-05851-x

**Published:** 2022-12-16

**Authors:** Bernhard Radlinger, Claudia Ress, Sabrina Folie, Karin Salzmann, Ana Lechuga, Bernhard Weiss, Willi Salvenmoser, Michael Graber, Jakob Hirsch, Johannes Holfeld, Christian Kremser, Patrizia Moser, Gabriele Staudacher, Tomas Jelenik, Michael Roden, Herbert Tilg, Susanne Kaser

**Affiliations:** 1 Christian Doppler Laboratory for Metabolic Crosstalk, Medical University Innsbruck, Innsbruck, Austria; 2 Department of Internal Medicine I, Medical University Innsbruck, Innsbruck, Austria; 3 Innpath GmbH, Innsbruck, Austria; 4 Institute of Zoology and Center of Molecular Biosciences Innsbruck (CBMI), Leopold Franzens University Innsbruck, Innsbruck, Austria; 5 Department of Cardiac Surgery, Medical University Innsbruck, Innsbruck, Austria; 6 Department of Radiology, Medical University Innsbruck, Innsbruck, Austria; 7 Institute for Clinical Diabetology, German Diabetes Center, Leibniz Center for Diabetes Research at Heinrich-Heine-University Düsseldorf, Düsseldorf, Germany; 8 Department of Endocrinology and Diabetology, Medical Faculty and University Hospital Düsseldorf, Heinrich-Heine-University Düsseldorf, Düsseldorf, Germany; 9 German Center for Diabetes Research, Partner Düsseldorf, München-Neuherberg, Germany

**Keywords:** Empagliflozin, Insulin resistance, Obesity, SGLT2 inhibition, Skeletal muscle mitochondria, Steatosis, Western-type diet

## Abstract

**Aims/hypothesis:**

Sodium–glucose cotransporter 2 (SGLT2) inhibitors are widely used in the treatment of type 2 diabetes, heart failure and chronic kidney disease. Their role in the prevention of diet-induced metabolic deteriorations, such as obesity, insulin resistance and fatty liver disease, has not been defined yet. In this study we set out to test whether empagliflozin prevents weight gain and metabolic dysfunction in a mouse model of diet-induced obesity and insulin resistance.

**Methods:**

C57Bl/6 mice were fed a western-type diet supplemented with empagliflozin (WDE) or without empagliflozin (WD) for 10 weeks. A standard control diet (CD) without or with empagliflozin (CDE) was used to control for diet-specific effects. Metabolic phenotyping included assessment of body weight, food and water intake, body composition, hepatic energy metabolism, skeletal muscle mitochondria and measurement of insulin sensitivity using hyperinsulinaemic–euglycaemic clamps.

**Results:**

Mice fed the WD were overweight, hyperglycaemic, hyperinsulinaemic and insulin resistant after 10 weeks. Supplementation of the WD with empagliflozin prevented these metabolic alterations. While water intake was significantly increased by empagliflozin supplementation, food intake was similar in WDE- and WD-fed mice. Adipose tissue depots measured by MRI were significantly smaller in WDE-fed mice than in WD-fed mice. Additionally, empagliflozin supplementation prevented significant steatosis found in WD-fed mice. Accordingly, hepatic insulin signalling was deteriorated in WD-fed mice but not in WDE-fed mice. Empagliflozin supplementation positively affected size and morphology of mitochondria in skeletal muscle in both CD- and WD-fed mice.

**Conclusions/interpretation:**

Empagliflozin protects mice from diet-induced weight gain, insulin resistance and hepatic steatosis in a preventative setting and improves muscle mitochondrial morphology independent of the type of diet.

**Graphical abstract:**

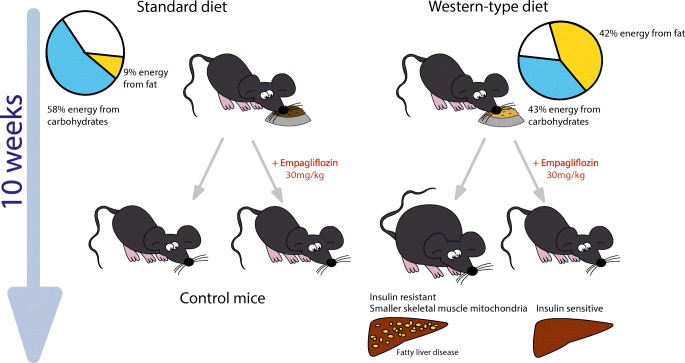

**Supplementary Information:**

The online version contains peer-reviewed but unedited supplementary material available at 10.1007/s00125-022-05851-x.

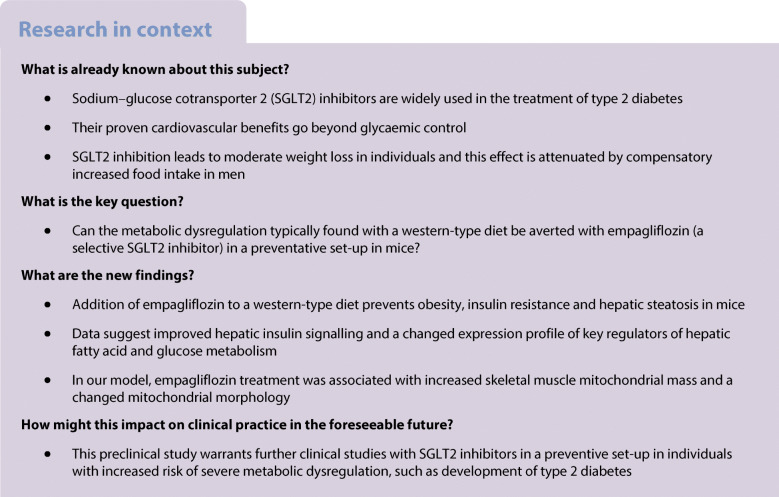



## Introduction

Obesity prevalence is dramatically increasing worldwide. Recently, overweight and obesity were estimated to account for 4 million deaths globally every year [1]. According to the WHO a vast 1.9 billion adults were overweight in 2016 and over 650 million were obese [2]. Chronic overfeeding, wrong choice of diet and an increasingly sedentary lifestyle are accepted as central contributing factors to the obesity pandemic [3]. There is a long and well-documented epidemiological and pathophysiological relationship between obesity and risk of type 2 diabetes. Despite extensive knowledge of the positive effects of a Mediterranean diet and physical activity on energy and glucose metabolism, sedentary lifestyle and intake of high-energy, fat- and sucrose-rich diets are still predominant in the western world [4].

Sodium–glucose cotransporter 2 (SGLT2) inhibitors are now widely used for their specific beneficial effects in individuals with type 2 diabetes [5, 6]. Beside their glucose-lowering properties these effects include cardio- and nephroprotection, and a modest reduction in BP and body weight [7, 8]. Interestingly, a weight loss of roughly 2–4 kg is usually only seen within the first 3 months of treatment despite ongoing glucosuria, probably explained by a compensatory increase in food intake [9]. Besides direct effects on hepatic inflammation, reactive oxygen species generation and mitochondrial function SGLT2 inhibitor-induced weight reduction might also contribute to beneficial effects on non-alcoholic fatty liver disease (NAFLD) [10, 11].

While SGLT2 inhibitors are well established in the treatment of type 2 diabetes, heart failure and chronic kidney disease, their role in the primary prevention of metabolic dysfunction is unclear. Prospective studies utilising SGLT2 inhibitors in the primary prevention of obesity are scarce and to our knowledge have not been conducted in humans. In preclinical studies, empagliflozin, a specific SGLT2 inhibitor, showed increased energy expenditure, browning of adipose tissue and decreased adipose tissue inflammation in mice fed a high-fat diet [12].

In this study we set out to test whether empagliflozin prevents weight gain and metabolic dysfunction in a mouse model of diet-induced obesity and insulin resistance.

## Methods

### Animals

A total of 140 male 6-week-old C57Bl/6 mice (Charles River Laboratories, Germany) were used in this study. Mice were kept under standard conditions of 12 h night–day cycle, 23±2–3°C and controlled humidity. After 1 week of acclimatisation, mice were fed four different diets ad libitum (SSNIFF Spezialdiäten, Germany): standard control diet (CD) (SSNIFF art. no. 1534-00); CD with added empagliflozin (CDE); western-type diet (WD) (21.2% fat and 33.3% sugar content, corresponding to 42% energy from fat and 43% energy from carbohydrates; SSNIFF art. no. E15721-34); and WD with added empagliflozin (WDE). Empagliflozin was provided by Boehringer Ingelheim and added directly to diets by SSNIFF to aim at an empagliflozin dose of 30 mg/kg body weight (260 mg empagliflozin/1000 g CD, 192 mg empagliflozin/1000 g WD). Dosing was based on previously published studies [13–15]. Group allocation was chosen randomly.

Diets were fed to the mice for 10 weeks, with weekly measurements of capillary blood glucose via tail vein puncture and a handheld glucometer (Accu-Chek Performa Nano; Roche, Switzerland) and weekly measurements of body weight and water and food consumption. Before weekly measurements of blood glucose, mice were fasted for 6 h. At the end of study mice were anaesthetised after a 6 h fast. Mice were killed via a combination of central blood collection with cardiac puncture and cervical dislocation. Tissue samples were collected immediately afterwards, snap-frozen in liquid nitrogen and stored until further processing at −80°C. All animal procedures were performed in accordance with the guidelines of the Austrian Animal Testing Act of 1988. Approval for this animal study was granted by the Austrian Federal Ministry for Education, Science and Research (application no. *BMWF-66.011/0066/ − V/3b/2018).*

### Hyperinsulinaemic–euglycaemic clamp

After 10 weeks of diet and/or treatment with empagliflozin, a silicone catheter was surgically inserted into the right external jugular vein of the mice to provide i.v. access as described before [16]. The catheter was connected to an i.v. access point placed behind the neck of the mouse (VABM1BSM/25; Instech Laboratories, USA). During surgery, mice were anaesthetised using a combination of breathable isoflurane (5% [vol./vol.] for induction of anaesthesia and 2–3% [vol./vol.] as anaesthesia maintenance; Zoetis, USA) and given an s.c. injection of piritramide (0.1 mg/kg; Piramal, India). Mice were kept at 37°C via a heating pad during surgery. At 3–5 days after surgery, mice had regained their pre-surgery weight (±10%) and hyperinsulinaemic–euglycaemic clamping was performed as described [16]. In short, after a 6 h fast, i.v. access was established via connecting the i.v. access point. Capillary blood glucose levels were measured using the cut tail method and a handheld glucometer during the experiment. Readings were taken with mice in the fasted state, during the set-up phase, and every 5–10 min during the clamp until stable euglycaemia was reached with a glucose target of 5.55–6.66 mmol/l (Fig. 3e and f). Insulin (insulin aspart; Novo Nordisk, Denmark) was infused steadily at 8 mU kg^−1^ min^−1^. A variable infusion of 20% (wt/vol.) glucose (Merck, USA) was given to reach and maintain the set glucose target.

At the end of the clamp, mice were anaesthetised and killed via cervical dislocation. Tissue and blood samples from these mice were not used for further analysis due to likely interference of the clamp experiment with subsequent analysis (especially analysis of the insulin signalling pathway).

### MRI studies

**Fig. 1:**
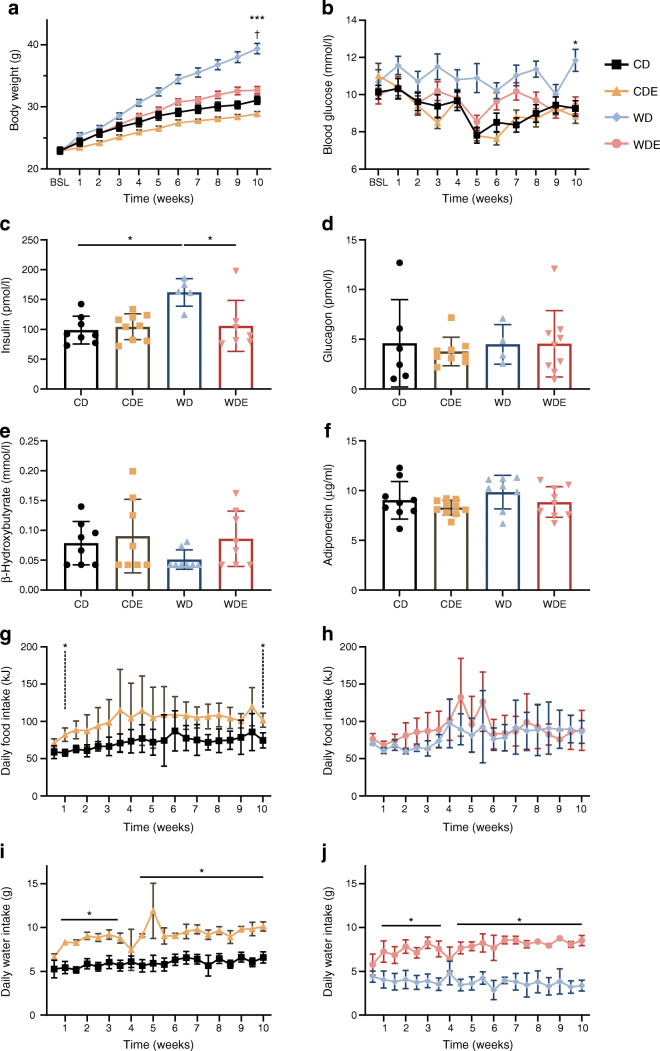
Characteristics of mice during the study. (**a**) Body weight of mice over the course of the study (mean ± SEM, *n*=16 [WD=15]). Two-way repeated measures ANOVA was used (*p* for diet effects, *p*<0.0001; *p* for diet × time interaction, *p*<0.0001). ***p<0.001 WD vs WDE; ^†^*p*<0.01 CD vs CDE at week 10. (**b**) Blood glucose levels during the study (mean ± SEM, *n*=16 [WD=15]). Two-way repeated measures ANOVA was used (*p* for diet effects <0.0001; *p* for diet × time interaction <0.0502). *p<0.05 WD vs WDE at week 10. (**c**–**f**) Plasma insulin (*n*=5–9) (**c**), glucagon (*n*=4–9) (**d**), β-hydroxybutyrate (*n*=8) (**e**) and adiponectin (*n*=8–10) (**f**) levels at week 10. (**g**, **h**) Daily food intake in CD and CDE mice (**g**) and WD and WDE mice (**h**) (*n*=4). (**i**, **j**) Daily water intake in CD and CDE mice (**i**) and WD and WDE mice (**j**) (*n*=4). Unless otherwise specified, data are presented as mean ± SD. Kruskal–Wallis test was performed for (**c**–**f**). Two-way ANOVA was performed for (**a**, **b**) and (**g**–**j**). Bars and asterisks (*p<0.05) indicate respective post hoc analysis. The key applies to (**a**, **b** and **g**–**j**). BSL, baseline

MRI studies were performed after the clamp studies in a subset of mice (*n*=6) using a 3T MRI scanner (Magnetom Skyra; Siemens, Germany). Transverse relaxation time (T2)-weighted Dixon Turbo Spin Echo sequences were acquired via coronal plane sections covering the whole mouse volume. T2-weighted fat- and water-separated images were generated. Total body volume and total fat volume was calculated using Fiji/ImageJ [17] and employing threshold-based segmentation with manual corrections (electronic supplementary material [ESM] Fig. 1). Fat depots were separated according to their anatomical location into three different compartments: subcutaneous fat; visceral fat; and retroperitoneal fat. Treatment groups were blinded in the data for quantitative analysis.

### Liver histology

Liver tissue samples were fixed using a standard formaldehyde fixation/paraffin embedding protocol. Samples were cut in sections and routine H&E staining was done. Grading of steatosis was done on five fields of view per sample in a blinded manner. A standard steatosis grading system was applied according to the percentage of lipid droplets (score: 0, <5%; 1, 5–33%; 2, 33–66%, 3, >66%) [18].

### Triacylglycerol and glycogen measurement

Hepatic triacylglycerol and glycogen levels were measured using an automated analyser (Hitachi Cobas C 311; Roche, Switzerland) with commercially available kits. For analysis of hepatic glycogen, tissue samples were weighed and minced, and glycogen was hydrolysed using 2 mol/l HCl at 100°C for 1 h. After pH neutralisation and centrifugation (10,000 *g* for 10 min), glucose was measured in the supernatant fraction. NaOH treatment was used as negative controls.

### Quantitative PCR and immunoblotting

Homogenisation of tissue samples, RNA isolation, quantification and reverse transcription was done as described previously in detail [13]. Quantitative PCRs were run on a 7900 HT Fast Real-Time cycler (Applied Biosystems, USA). Hypoxanthine guanine phosphoribosyl transferase (encoded by *Hprt*) mRNA expression was used for normalisation of data. All quantitative PCRs were performed with two technical replicates. Western blotting was performed as described previously [13].

### Citrate synthase assay

Citrate synthase activity in tissue samples was determined using a commercially available kit (Sigma-Aldrich, USA). Protein lysate was freshly prepared before the experiment. Oxaloacetate and acetyl-CoA were added to the samples. Formation of free -SH groups was photometrically measured using a shift in absorption via 5,5′-dithiobis-(2-nitrobenzoic acid)/2-nitro-5-thiobenzoic acid (DTNB/TNB) at 512 nm over the course of 2 min. Baseline activity was subtracted for subsequent analysis. Analysis was performed with two technical replicates. Protein concentration of samples were used for normalisation of data.

### Transmission electron microscopy

Preparation of specimens for transmission electron microscopy (TEM) was done according to a previously published protocol [13]. For fixation, Karnovsky fixative (2% [wt/vol.] paraformaldehyde, 2.5% [wt/vol.] glutaraldehyde) was used. TEM sampling was done in three mice per group. Image magnification was 3200×; one image covered an area of 8.87×8.87 μm. Skeletal muscle fibre types were differentiated into type I and type II fibres using both Z-plane thickness and intramyofibrillary mitochondrial content and appearance based on previously published work [19, 20]. Only longitudinal sections were used for further analysis. Using this approach, 220 images, with a total of 9453 mitochondria, were collected for subsequent analysis. Fiji/ImageJ [17] was used for quantitative analysis of mitochondrial morphology. Group allocation was blinded before subsequent analysis.

### Laboratory analysis

Plasma levels of insulin, glucagon and adiponectin were measured using commercially available ELISAs according to the manufacturer’s instructions (respectively, Ultra Sensitive Mouse Insulin ELISA Kit [Crystal Chem, USA], Glucagon ELISA [10 μl kit; Mercodia, Sweden] and Mouse Adiponectin ELISA Kit [R&D Systems, USA]). β-Hydroxybutyrate levels were UV photometrically measured using an available kit (Ketonebody Assay Kit; Sigma-Aldrich, USA).

### Statistical analysis

GraphPad Prism 8.4.1 was used for statistical analysis (GraphPad Software, USA). ANOVA analysis was performed according to assumptions and normality of residuals was checked using the Kolmogorov–Smirnov test. For skewed residuals, the non-parametric Kruskal–Wallis test was used. Post hoc group comparisons were performed with pre-specified comparisons (CD vs WD, CD vs CDE, WD vs WDE) and either Sidak’s or Dunn’s method were used to correct for multiple testing. Systemic data from the feeding period (body weight, blood glucose, water and food intake) were analysed via a two-way ANOVA model with Geisser–Greenhouse correction. To address potential confounding in the analysis of TEM data, multiple linear regression was performed using R Version 4.0.2 and RStudio Version 1.4.615 [21]. Statistical significance was conferred with *p* values <0.05. Unless otherwise stated, data are expressed as means ± SD. For highly skewed data (mitochondrial data from TEM analysis), boxplots were used to present data.

## Results

### Empagliflozin treatment prevents diet-induced obesity, hyperglycaemia and hyperinsulinaemia

**Fig. 2:**
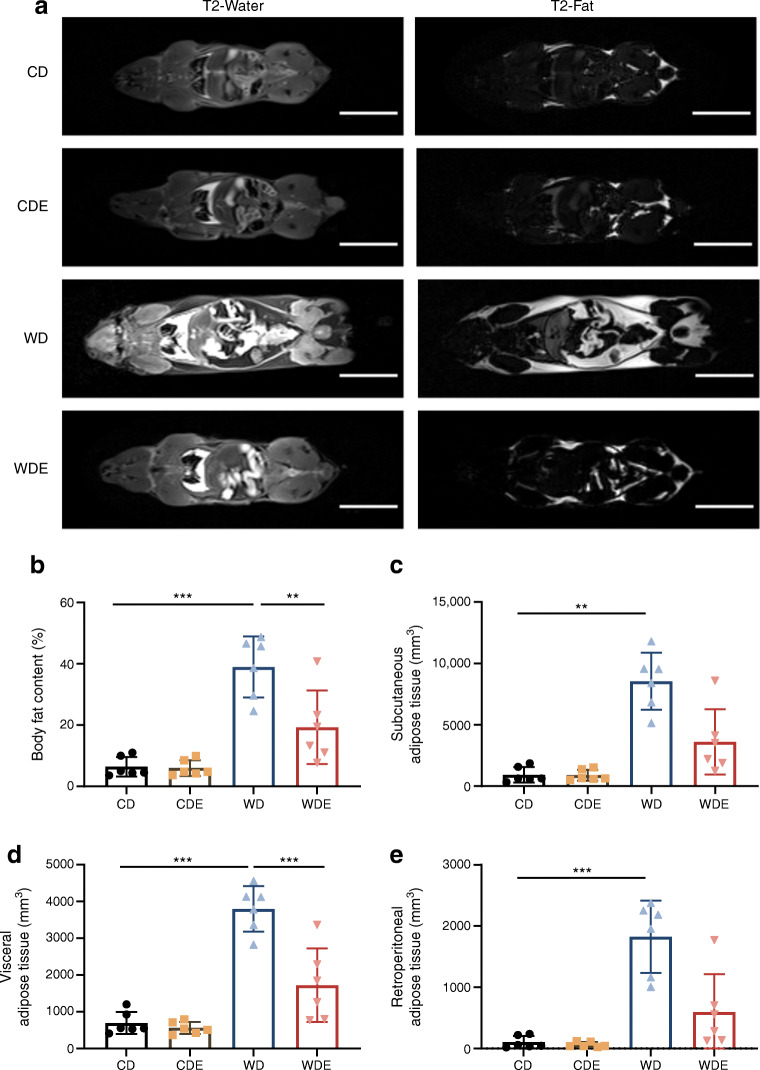
MRI analysis of total body fat and fat distribution in mice. (**a**) Representative coronal sections of 3T MRI studies. Water- and fat-separated images generated by the T2-Weighted Dixon Turbo Spin Echo sequence are shown. Scale bar, 20 mm. (**b**) Quantification of body fat content as per cent of total body volume (also calculated from MRI sequences) (*n*=6). (**c**–**e**) Different adipose tissue compartments in mm^3^: subcutaneous (**c**) (*n*=6); visceral (**d**) (*n*=6) and retroperitoneal (**e**) fat depot (*n*=6). Data are presented as mean ± SD. ANOVA was performed for (**b**, **d**). Kruskal–Wallis test was performed for (**c**, **e**). Bars and asterisks (**p<0.01, ***p<0.001) indicate respective post hoc analysis

After 10 weeks on the diets, WD-fed mice had a significantly higher body weight than CD-fed mice. Treatment with empagliflozin prevented diet-induced weight gain during the study, resulting in comparable body weights for WDE-fed and CD-fed mice (Fig. 1a). Accordingly, WDE-fed mice displayed a significantly lower total body fat content and smaller subcutaneous, visceral and retroperitoneal adipose tissue depots when compared with WD-fed mice (Fig. 2). WD-fed mice had elevated fasting blood glucose levels during the study period, with the difference being significant at week 10 when compared with CD-fed mice (Fig. 1b). Empagliflozin prevented hyperglycaemia as well as the accompanying hyperinsulinaemia in WDE-fed mice (Fig. 1b, c). Glucagon, ketone body (β-hydroxybutyrate) and adiponectin levels were comparable between all groups (Fig. 1d–f). Empagliflozin treatment did not significantly affect daily food intake in CD-fed or WD-fed mice (Fig. 1g, h). Water intake was significantly increased upon addition of empagliflozin to the diet, irrespective of whether mice were fed CD or WD (Fig. 1i, j). Glucosuria was seen in all mice upon empagliflozin treatment, irrespective of the type of diet (ESM Fig. 2).

### Empagliflozin treatment prevents diet-induced insulin resistance

**Fig. 3:**
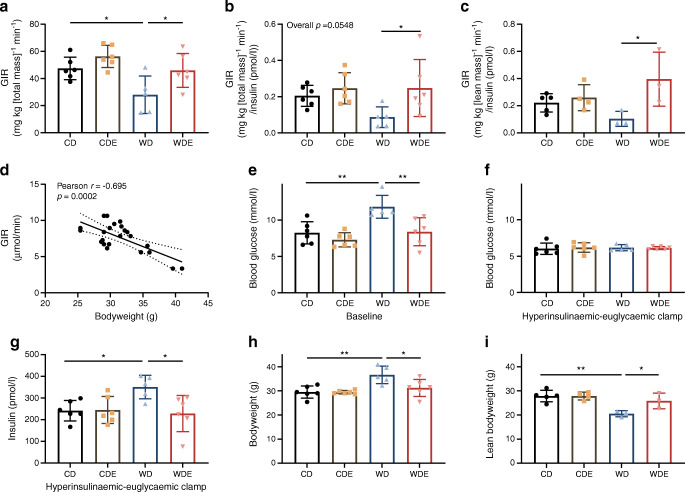
Hyperinsulinaemic–euglycaemic clamp studies. (**a**) GIR at steady-state euglycaemic clamp conditions (*n*=5 or 6). (**b**) GIR per total body weight adjusted for insulin concentration at clamp conditions (*n*=5 or 6). (**c**) GIR per lean body weight adjusted for insulin concentration at clamp conditions (*n*=3–5). (**d**) Correlation of GIR (unadjusted for body weight, μmol/min) and body weight (*n*=23). (**e**, **f**) Blood glucose at baseline (beginning of clamp study) (**e**) and at hyperinsulinaemic–euglycaemic conditions (**f**) (*n*=5 or 6). (**g**) Plasma insulin levels at clamp conditions (*n*=5 or 6). (**h**, **i**) Total (**h**) (*n*=5 or 6) and lean (**i**) (*n*=3–5) bodyweight. Data are presented as mean ± SD. ANOVA was performed for (**a**–**c**) and (**e**–**i**). Bars and asterisks (*p<0.05, **p<0.01) indicate respective post hoc analysis

After 10 weeks of being fed the diets, the whole-body insulin sensitivity of the mice was measured using hyperinsulinaemic–euglycaemic clamps. The glucose infusion rate (GIR) required to maintain stable euglycaemia under hyperinsulinaemic conditions was found to be significantly higher in WDE-fed mice than in WD-fed mice, indicating increased insulin sensitivity (Fig. 3a). Remarkably, the mean GIR (and thus insulin sensitivity) was comparable in WDE- and CD-fed mice. When differences in plasma insulin concentration under clamp conditions and differences in lean body mass of mice were taken into consideration, there was an even greater difference seen when comparing insulin sensitivity in WD and WDE mice (Fig. 3b, c). The GIR was strongly correlated with body weight, suggesting that lack of excess weight gain significantly contributed to the improved insulin sensitivity in WDE mice (Fig. 3d).

### Empagliflozin provides protection against hepatic steatosis and improves hepatic insulin signalling

**Fig. 4:**
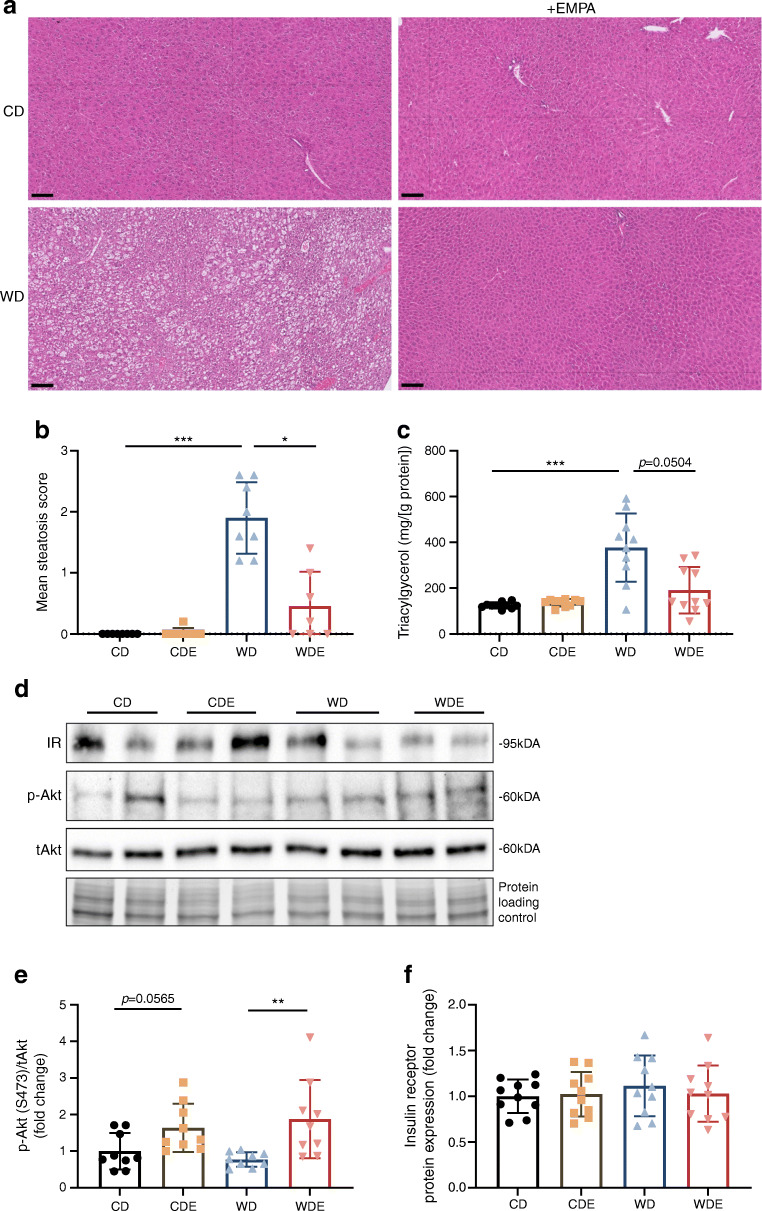
Histological and metabolic assessments of liver specimen after 10 week of diets. (**a**) Representative H&E stains of liver sections after 10 weeks of respective diets. Scale bar, 100 μm. (**b**) Mean steatosis score assessed in a blinded manner (*n*=7 or 8). (**c**) Liver triacylglycerol content (*n*=10). (**d**) Representative western blots of hepatic insulin signalling. (**e**, **f**) Corresponding densitometry of p-Akt/tAkt (**e**) (*n*=9) and insulin receptor (**f**) (*n*=9). See ESM Figs 4 and 5 for full-length western blots. Data are presented as mean ± SD. Kruskal–Wallis test was performed for (**b**, **c**, **e**). ANOVA was performed for (**f**). Bars and asterisks (*p<0.05, **p<0.01, ***p<0.001) indicate respective post hoc analysis

The grade of steatosis was significantly lower in WDE-fed mice than in WD-fed mice and measurement of intrahepatic lipid content corroborated histological findings (Fig. 4a–c). Accordingly, hepatic insulin signalling as assessed by phosphorylated Akt/total Akt (p-Akt/tAkt) was significantly higher in WDE-fed mice than in WD-fed mice (Fig. 4d, e). Levels of insulin receptor expression were comparable between all groups (Fig. 4d, f).

### Empagliflozin supplementation affects hepatic energy metabolism

**Fig. 5:**
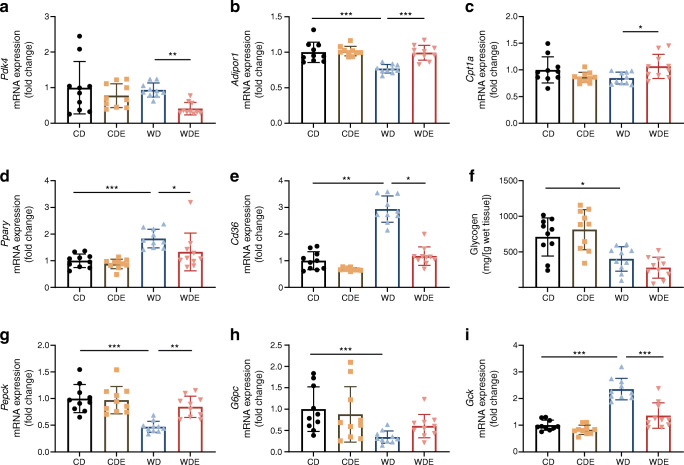
Liver expression analysis of key factors of hepatic insulin sensitivity and fatty acid metabolism. (**a**–**e**) *Pdk4* (**a**) (*n*=10), *Adipor1* (**b**) (*n*=10), *Cpt1a* (**c**) (*n*=10), *Pparγ* (**d**) (*n*=10) and *Cd36* (**e**) (*n*=10) mRNA levels. (**f**) Glycogen content of liver samples (*n*=10). (**g**–**i**) *Pepck* (**g**) (*n*=10), *G6pc* (**h**) (*n*=10) and *Gck* (**i**) (*n*=10) mRNA levels. Data are presented as mean ± SD. Kruskal–Wallis test was performed for (**a**, **c**–**e**, **g**, **h**). ANOVA was performed for (**b**, **f**, **i)**. Bars and asterisks (*p<0.05, **p<0.01, ***p<0.001) indicate respective post hoc analysis

mRNA expression of *Pdk4*, encoding pyruvate dehydrogenase kinase 4 (PDK4, an isoform known to regulate hepatic insulin signalling and energy metabolism), was significantly lower in the liver of WDE-fed mice compared with WD-fed mice (Fig. 5a). Increased expression of *Adipor1* (encoding adiponectin receptor isoform I) and *Cpt1a* (encoding carnitine palmitoyltransferase I) mRNA suggested increased β-oxidation in livers of WDE-fed mice compared with WD-fed mice (Fig. 5b, c). On the other hand, liver expression of *Pparγ* (also known as *Pparg*, encoding peroxisome proliferator-activated receptor γ) and *Cd36* mRNA was lower in WDE-fed mice than in WD-fed mice, suggesting lower hepatic fatty acid uptake (Fig. 5d, e). Glycogen levels (Fig. 5f) were not affected by empagliflozin, irrespective of the type of diet, while *Pepck* (encoding PEPCK) and *G6pc* (encoding glucose-6 phosphatase) mRNA levels were increased in WDE-fed mice when compared with WD-fed mice, indicative of higher gluconeogenesis (Fig. 5g, h). *Gck* (encoding glucokinase) mRNA expression was significantly increased in WD-fed mice compared with CD-fed mice (Fig. 5i); addition of empagliflozin to the WD led to a normalisation of *Gck* mRNA expression.

### Empagliflozin treatment affects mitochondrogenesis in skeletal muscle

**Fig. 6:**
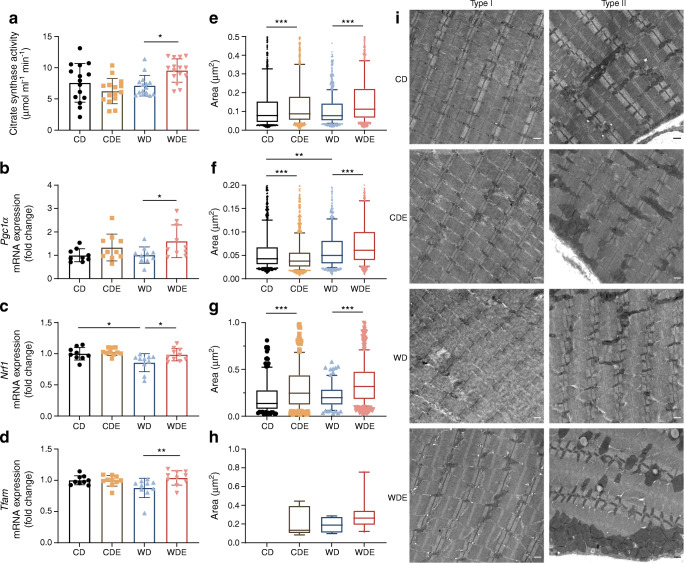
Mitochondrial biogenesis and morphology in skeletal muscle. (**a**) Citrate synthase activity. (**b**–**d**) *Pgc1α* (**b**) (*n*=15), *Nrf1* (**c**) (*n*=10) and *Tfam* (**d**) (*n*=10) mRNA expression levels. (**e**–**h**) Quantification of mitochondrial area in different fibre types and different subcellular location in skeletal muscle fibres. (**e**) (*n*=785–1370); type I fibres and intramyocellular location (**f**) (*n*=812–1213); type II fibres and subsarcolemmal location (**g**) (*n*=81–457); and type I fibres and subsarcolemmal location (**h**) (*n*=0–15). (**i**) Representative TEM sections of different skeletal muscle fibre types. Different size and morphological features of mitochondria of different subcellular compartments (intramyocellular/subsarcolemmal) are best seen in type II fibres in WDE-fed mice. Scale bar, 500 nm. Data are presented as mean ± SD (**a**–**d**) and as boxplots (10th–25th percentiles, median and 75th–90th percentiles are shown) (**e**–**h**). ANOVA was performed for (**a**–**c**). Kruskal–Wallis test was performed for (**d**–**g**). Bars and asterisks (*p<0.05, **p<0.01, ***p<0.001) indicate respective post hoc analysis

Citrate synthase activity, as a biomarker of cumulative mitochondrial mass, was increased by addition of empagliflozin to the WD (Fig. 6a). In line, expression levels of *Pgc1α* (also known as *Ppargc1a*, encoding peroxisome proliferator-activated receptor γ coactivator 1-α [PGC1α]) mRNA as well as *Nrf1* (encoding nuclear respiratory factor 1) and *Tfam* (encoding mitochondrial transcription factor A) mRNA were increased in WDE-fed mice when compared with WD-fed mice (Fig. 6b–d). Irrespective of the type of diet, empagliflozin supplementation was associated with alterations of subsarcolemmal and intermyofibrillar mitochondrial size and morphology in both types of skeletal muscle fibres as assessed by TEM (Fig. 6e–h). Dietary empagliflozin supplementation led to larger mitochondria and significant changes in the aspect ratio and circularity of mitochondria (ESM Table 1). Skeletal muscle fibre type and myocellular location of mitochondria are known to affect size and shape of mitochondria. To account for this confounding effect, we performed multiple linear regression analysis after adjusting for fibre type and subcellular localisation, and confirmed a drug-specific effect (ESM Table 1). Compared with liver tissue, changes in the p-Akt/tAkt ratio were modest in skeletal muscle (ESM Fig. 3a, b). Skeletal muscle triacylglycerol content was higher in WD-fed mice than in WDE-fed mice, without the difference reaching statistical significance (ESM Fig. 3c). Skeletal muscle glycogen content was unchanged with addition of empagliflozin (ESM Fig. 3d)

## Discussion

SGLT2 inhibitors have an indispensable role in the treatment of type 2 diabetes, chronic kidney disease and heart failure. Despite clinical evidence on cardio- and renoprotection the underlying beneficial mechanisms are still under discussion and include moderate weight loss, haemodynamic changes and cellular effects. While the glucose-lowering, cardioprotective and renoprotective properties of SGLT2 inhibitors are well established and these drugs are widely used in clinical practice, it is not clear whether SGLT2 inhibitors are capable of preventing metabolic disease in a high-risk scenario.

The aim of this study was to investigate whether SGLT2 inhibition provides protection against diet-induced weight gain, disturbances in glucose metabolism and fatty liver disease. The study was performed in male C57BL/6 mice on a WD as this setting is known to induce a distinct phenotype of metabolic disease including obesity, insulin resistance and hepatic steatosis [22].

Various mechanisms, especially in relation to possible benefits of SGLT2 inhibition regarding fatty liver disease, have been proposed and reviewed before [10, 23]. Mechanisms include attenuation of inflammation and macrophage polarisation [12, 24], autophagy [25], endoplasmic reticulum stress [25–27], and attenuation of steatosis and fibrosis [28] to name a few examples. However, whether SGLT2 inhibition is capable of preventing the development of obesity and insulin resistance is currently poorly studied. Therefore we chose to study empagliflozin in a diet-induced mouse model and to carry out extensive metabolic phenotyping to assess the metabolic health of the mice.

Adipose tissue plays a key role in the pathophysiology of insulin resistance, type 2 diabetes and fatty liver disease mainly via the release of NEFA, proinflammatory cytokines and adipocytokines [29, 30]. In our study, we show that empagliflozin supplementation prevents excess weight gain in WD-fed mice while it had no relevant effect on adipose tissue depots in CD-fed mice. MRI studies revealed that empagliflozin supplementation reduced diet-induced expansion of subcutaneous, visceral and retroperitoneal adipose tissue depots. In a previous study by Vallon et al [31], epididymal fat adipocyte size was found to be reduced upon empagliflozin treatment in C57BL/6-background mice, while increased adipocyte size was found in insulin-deficient Akita mice.

In individuals with type 2 diabetes, SGLT2 inhibitor treatment is associated with a modest weight reduction, which is typically seen during the initial phase of treatment. As expected, in our study empagliflozin-treated mice displayed marked glycosuria irrespective of type of diet. The weight difference between empagliflozin-treated and control mice might be explained by urinary energy loss and increased energy expenditure upon SGLT2 inhibitor treatment, as reported previously [12]. Glucose excretion into the urine was measured in fasted mice only in this study. However, a contribution by urinary glucose excretion in empagliflozin-treated mice to overall GIR in mice from the hyperinsulinaemic–euglycaemic clamp part of the study cannot be ruled out by the study design.

Interestingly, in humans, the initial weight loss is attenuated by a compensatory increase in energy intake [9]. In previous murine studies, both unchanged and increased food intake upon empagliflozin treatment was reported [31–33]. In our study, empagliflozin supplementation was associated with increased food intake in CD- and WD-fed mice, without the difference reaching statistical significance, as estimated by twice weekly determination of the food remaining in each cage. However, the sensitivity of this method might be too low to detect small differences in food intake.

Importantly, in our model, empagliflozin protected the mice against WD-induced insulin resistance as shown by hyperinsulinaemic–euglycaemic clamp studies. When adjusting pure GIRs for lean body mass and increased insulin levels at clamp conditions the results indicate an even more drastic increase in insulin sensitivity upon addition of empagliflozin to WD. A full dataset for lean body mass, clamp data and insulin levels was only available in a small sample size, so results should be seen as hypothesis-generating despite being statistically significant.

We found increased hepatic Akt phosphorylation, indicating increased hepatic insulin sensitivity with dietary empagliflozin supplementation. Akt phosphorylation in WD-fed compared with CD-fed mice was similar, although fasting insulin plasma levels were not. Mice on the WD were hyperinsulinaemic compared with CD-fed mice and this might explain similar Akt phosphorylation despite the presence of insulin resistance.

Body weight is a major contributing factor to whole-body insulin sensitivity, with some authors arguing it is the most important predicting factor especially after weight loss [34, 35]. Here, we corroborate this association by correlating body weight with GIR (Fig. 3d). A limitation of the study is the relative per-body-weight dose adjustment of insulin for the clamp procedure instead of adjustment for lean body mass.

Diet-induced obesity and insulin resistance are strongly associated with NAFLD. NAFLD is commonly associated with hepatic insulin resistance and results in further deterioration of systemic glucose metabolism [36, 37]. In our study, empagliflozin protected the WD-fed mice against diet-induced hepatic steatosis. In obese people with moderately controlled type 2 diabetes, empagliflozin treatment for 20 weeks resulted in improved blood glucose control, moderate weight reduction and significant improvement in liver triacylglycerol content [38]. In previous studies, significant weight loss of greater than 10% of initial body weight led to the resolution of steatosis in individuals with obesity [36, 37]. A study by Kahl et al showed that empagliflozin reduces liver fat content in individuals with well-controlled type 2 diabetes [11]. Interestingly, in that study no change in insulin sensitivity was observed, in contrast to findings of improved insulin sensitivity with empagliflozin in our study. In obese, insulin-resistant C57BL/6J mice Xu and colleagues reported that empagliflozin diminished weight gain and reduced deteriorations of insulin sensitivity and hepatic steatosis of ongoing high-fat-diet feeding [12, 24]. Mechanistically, increased energy expenditure and browning of adipose tissue as well as reduced inflammation in adipose tissue and the liver were found upon SGLT2 inhibition.

Here we show that empagliflozin not only exerts beneficial effects on overt NAFLD but also protects against diet-induced hepatic steatosis. Interestingly, we found reduced *Pdk4* mRNA expression in WDE-fed mice. Hepatic PDK4 expression has been linked to impaired insulin sensitivity and fatty liver disease [39–41] through stimulation of fatty acid uptake and synthesis in the liver. Accordingly, in our study reduced *Pdk4* expression was accompanied by decreased fatty acid uptake, estimated by expression of key regulators of cellular fatty acid metabolism. Barres et al [42] reported altered promotor methylation of PDK4 in obese individuals, with the alteration being restored by significant weight loss, suggesting that empagliflozin protects against obesity-induced alterations in PDK4 expression upon WD feeding.

The p-Akt/tAkt ratio was increased in WDE-fed mice compared with WD-fed mice, suggesting enhanced hepatic insulin sensitivity. While empagliflozin supplementation did not affect hepatic glycogen content, expression data suggest increased gluconeogenesis and decreased glycolysis in WDE-fed mice when compared with WD-fed mice. While increased gluconeogenesis and enhanced insulin signalling seems controversial, these data might be explained by the significantly reduced PDK4 expression in WDE-fed mice. PDK4 inhibits the pyruvate dehydrogenase complex, which links fatty acid and glucose metabolism by catalysing the oxidative decarboxylation of pyruvate [40, 43]. PDK4 deficiency was shown to prevent hepatic steatosis upon high-fat-diet feeding, probably due to increased PGC1⍺ activity. This decreased activity is associated with increased levels of PEPCK and reduced capacity for de novo fatty acid synthesis [44]. Importantly, PDK4 expression is increased and its methylation decreased in type 2 diabetes [45]. We hypothesise that decreased *Pdk4* mRNA expression might underlie the increased hepatic gluconeogenesis observed in WDE-fed mice when compared with WD-fed mice. It might be speculated that the reduced *Pdk4* mRNA expression in livers of WDE-fed mice might be associated with reduced oxidation of carbohydrates leading to a shift towards fatty acid oxidation and stimulation of gluconeogenesis. In contrast, reduced glycolysis, as indicated by decreased glucokinase mRNA expression levels, in WDE-fed mice in comparison with WD-fed mice might reflect improved hepatic insulin sensitivity.

Unexpectedly, expression markers of key enzymes of gluconeogenesis were decreased in WD-fed mice when compared with CD-fed mice, suggesting preserved insulin action in insulin resistance partially compensated by hyperinsulinaemia. Further dynamic tests will be necessary to better understand the effects of SGLT2 inhibitors on hepatic glycogen metabolism.

Mechanistically, high influx of adipose-tissue-derived fatty acids, diminished suppression of fatty acid synthesis in the liver, reduced hepatic insulin signalling, and impaired adiponectin signalling are major drivers of triacylglycerol accumulation in the liver. Our data suggest that empagliflozin supplementation reduces fatty acid uptake and synthesis (probably by affecting PDK4 expression), enhances adiponectin activity (due to increased adiponectin receptor expression) and improves hepatic insulin signalling (as shown by increasing Akt phosphorylation).

Besides liver and adipose tissue, glucose disposal of skeletal muscle is another major determinant of systemic insulin sensitivity. Here, we did not see relevant changes in p-Akt/tAkt ratio (ESM Fig. 3). However, assessment of Akt activation was performed in fasted mice, whereas data from clamp studies were acquired under hyperinsulinaemic conditions. In skeletal muscle, abnormal mitochondrial function is thought to play a key role in pathophysiology of cellular insulin resistance [46, 47].

Remarkably, our data suggest diet-independent effects on mitochondrial morphology and mass, as shown by increased citrate synthase activity and elevated mRNA expression levels of *Pgc1α* and downstream targets *Nrf1* and *Tfam* upon empagliflozin supplementation. Finally, ultrastructural analysis revealed skeletal muscle mitochondria to be larger and have a more rounded shape after empagliflozin supplementation compared with no supplementation. Altered skeletal muscle mitochondrial morphology is a hallmark of type 2 diabetes and obesity [48]. Our group and others have shown weight-independent beneficial effects of empagliflozin on mitochondrial morphology [13] and improved mitochondrial respiration in cardiac muscle [15]. Additionally, empagliflozin treatment improved skeletal muscle mitochondrial function in a murine model of heart failure [32].

Although previous studies have shown the beneficial effects of empagliflozin treatment in obese, insulin-resistant or diabetic humans or rodents, this is to our knowledge the first study that demonstrates preventative effects of empagliflozin in healthy mice. Our study suggests that empagliflozin treatment not only reverses obesity (or diabetes-specific alterations in metabolically important tissues such as adipose tissue, liver and skeletal muscle) but also protects against systemic and tissue-specific metabolic defects seen upon high-fat diet intake.

In conclusion, empagliflozin treatment protects mice against obesity, insulin resistance and hepatic steatosis in a setting of high-energy, high-sucrose and high-fat intake. Although many metabolic effects of empagliflozin might predominantly result from prevention of excess weight gain, our data suggest that additional diet-independent benefits on mitochondrial structure and mass in skeletal muscle might also contribute to preservation of systemic insulin sensitivity.

## Supplementary Information

ESM (PDF 1.03 MB)

## Data Availability

Original data are available upon reasonable request from the corresponding author.

## Abbreviations

CD: Standard control diet
CDE: CD + empagliflozin
GIR: Glucose infusion rate
NAFLD: Non-alcoholic fatty liver disease
PDK4: Pyruvate dehydrogenase kinase 4
PGC1α: Peroxisome proliferator-activated receptor γ coactivator 1-α
SGLT2: Sodium–glucose cotransporter 2
T2: Transverse relaxation time
TEM: Transmission electron microscopy
WD: Western-type diet
WDE: WD + empagliflozin
