# Supplementary material for: Empagliflozin protects mice against diet-induced obesity, insulin resistance and hepatic steatosis
Source: Diabetologia. 2022 Dec 16;66(4):754–67. doi: 10.1007/s00125-022-05851-x (PMC9947060; doi:10.1007/s00125-022-05851-x)
Supplement: Supplementary file 1 — (PDF 1.03 MB) [file 125_2022_5851_MOESM1_ESM.pdf]

*Electronic supplementary material*

# Empagliflozin protects mice against diet-induced obesity, insulin resistance and hepatic steatosis

Bernhard Radlinger[1,2], Claudia Ress[1,2], Sabrina Folie[1,2], Karin Salzmann[1,2], Ana Lechuga[1,2], Bernhard Weiss[1,2,3], Willi Salvenmoser[4], Michael Graber[5], Jakob Hirsch[5], Johannes Holfeld[5], Christian Kremser[6], Patrizia Moser[3], Gabriele Staudacher[1,2], Tomas Jelenik[7], Michael Roden[7,8,9], Herbert Tilg[2] and Susanne Kaser[1,2]

1. Christian Doppler Laboratory for Metabolic Crosstalk, Medical University Innsbruck, Innsbruck, Austria
2. Department of Internal Medicine I, Medical University Innsbruck, Innsbruck, Austria
3. Innpath GmbH, Innsbruck, Austria
4. Institute of Zoology and Center of Molecular Biosciences Innsbruck (CBMI), Leopold Franzens University Innsbruck, Innsbruck, Austria
5. Department of Cardiac Surgery, Medical University Innsbruck, Innsbruck, Austria
6. Department of Radiology, Medical University Innsbruck, Innsbruck, Austria
7. Institute for Clinical Diabetology, German Diabetes Center, Leibniz Center for Diabetes Research at Heinrich-Heine-University Düsseldorf, Düsseldorf, Germany
8. Department of Endocrinology and Diabetology, Medical Faculty and University Hospital Düsseldorf, Heinrich-Heine-University Düsseldorf, Düsseldorf, Germany
9. German Center for Diabetes Research, Partner Düsseldorf, München-Neuherberg, Germany

## Corresponding author

Susanne Kaser

E-mail: Susanne.Kaser@i-med.ac.at

Received: 5 September 2022 / Accepted: 31 October 2022

**ESM Table 1, Multiple Linear Regression Model regarding mitochondrial Area (µm²)(adjusted for type and fiber)**

Group = Standard control diet was used as reference group in the model below.

Type a = intramyofibrillar mitochondria (reference group in model)

Type b= subsarcolemmal mitochondria

Fiber w = Type I fiber (reference group in model)

Fiber r = Type II fiber

```
##
## Call:
## lm(formula = Area ~ group + type + fiber, data = TEM)
##
## Residuals:
##      Min       1Q   Median       3Q      Max
## -0.32823 -0.06908 -0.02546  0.02254  2.05533
##
## Coefficients:
##              Estimate Std. Error t value Pr(>|t|)
## (Intercept)  0.130929   0.003799  34.463  < 2e-16 ***
## groupCDE      0.012744   0.004311   2.956  0.00313 **
## groupWD      -0.008853   0.004704  -1.882  0.05985 .
## groupWDE      0.041971   0.004481   9.366  < 2e-16 ***
## typeb        0.173335   0.005060  34.255  < 2e-16 ***
## fiberw      -0.076466   0.003226 -23.705  < 2e-16 ***
## ---
## Signif. codes:  0 '***' 0.001 '**' 0.01 '*' 0.05 '.' 0.1 ' ' 1
##
## Residual standard error: 0.1475 on 9447 degrees of freedom
## Multiple R-squared:  0.2309, Adjusted R-squared:  0.2305
## F-statistic: 567.2 on 5 and 9447 DF,  p-value: < 2.2e-16
```

**Multiple Linear Regression Model regarding aspect ratio (adjusted for type and fiber). Aspect ratio = major axis/minor axis**

```
## Call:
## lm(formula = AR ~ group + type + fiber, data = TEM)
##
## Residuals:
##     Min      1Q  Median      3Q     Max
## -1.3652 -0.5661 -0.2068  0.3316  9.3198
##
## Coefficients:
##             Estimate Std. Error t value Pr(>|t|)
## (Intercept)  2.03883    0.02218  91.929  < 2e-16 ***
## groupCDE      0.02958    0.02517   1.175     0.24
## groupWD       0.37842    0.02746  13.781  < 2e-16 ***
## groupWDE      0.23658    0.02616   9.043  < 2e-16 ***
## typeb       -0.21335    0.02954  -7.222 5.50e-13 ***
```

```
## fiberw      -0.14916    0.01883  -7.921 2.62e-15 ***
## ---
## Signif. codes:  0 '***' 0.001 '**' 0.01 '*' 0.05 '.' 0.1 ' ' 1
##
## Residual standard error: 0.8609 on 9447 degrees of freedom
## Multiple R-squared:  0.03951,    Adjusted R-squared:  0.03901
## F-statistic: 77.73 on 5 and 9447 DF,  p-value: < 2.2e-16
```

**Multiple Linear Regression Model regarding circularity (adjusted for type and fiber).**

**Circularity = 4* π * (area/perimeter²)**

```
## Call:
## lm(formula = Circ. ~ group + type + fiber, data = TEM)
##
## Residuals:
##      Min       1Q   Median       3Q      Max
## -0.59799 -0.08496  0.02706  0.10801  0.27759
##
## Coefficients:
##              Estimate Std. Error t value Pr(>|t|)
## (Intercept)  0.752802   0.003700 203.466  < 2e-16 ***
## groupCDE    -0.024656   0.004199  -5.872 4.44e-09 ***
## groupWD     -0.088392   0.004581 -19.295  < 2e-16 ***
## groupWDE    -0.064036   0.004364 -14.673  < 2e-16 ***
## typeb        0.020983   0.004928   4.258 2.08e-05 ***
## fiberw       0.040191   0.003141  12.793  < 2e-16 ***
## ---
## Signif. codes:  0 '***' 0.001 '**' 0.01 '*' 0.05 '.' 0.1 ' ' 1
##
## Residual standard error: 0.1436 on 9447 degrees of freedom
## Multiple R-squared:  0.06703,    Adjusted R-squared:  0.06653
## F-statistic: 135.7 on 5 and 9447 DF,  p-value: < 2.2e-16
```

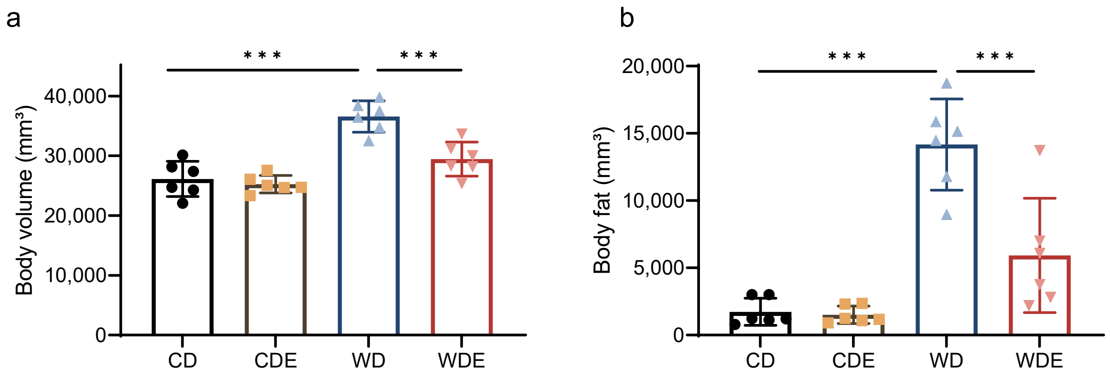


**ESM Fig. 1, Body volume and body fat of mice.** (**a**) Body volume in $mm^3$ and (**b**) total body fat in $mm^3$. ANOVA was performed for (**a**, **b**). Bars and asterisks (*p<0.05, **p<0.01, ***p<0.001) indicate respective post hoc analysis. Data are represented as mean ± SD.

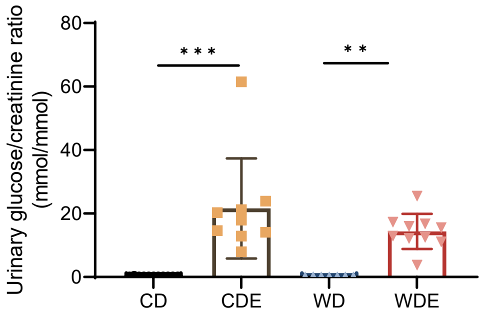


**ESM Fig. 2, Urinary glucose in spot urine after 10 weeks of treatment.** Spot urine at the end of the study (n=7-11). Kruskal-Wallis test was performed. Bars and asterisks (*p<0.05, **p<0.01, ***p<0.001) indicate respective post hoc analysis. Data are represented as mean ± SD.

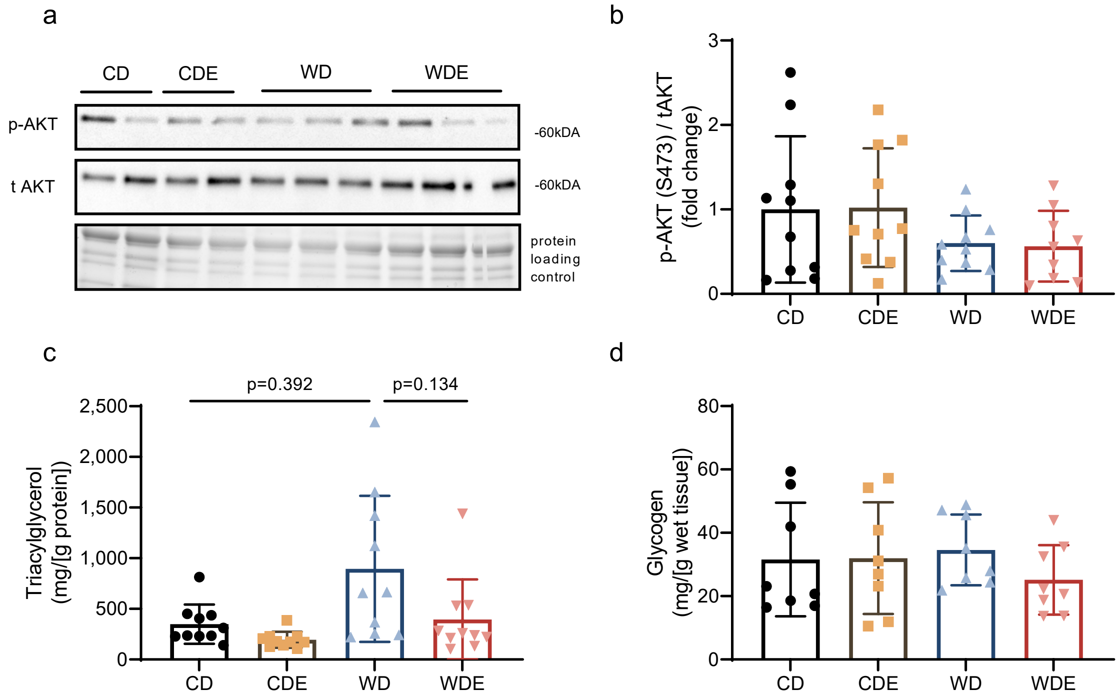


**ESM Fig. 3, Skeletal muscle insulin signalling.** (**a**) Representative western blots of p-AKT and tAKT. (**b**) Corresponding densitometry of p-Akt/tAKT (n=9 or 10). (**c**) Skeletal muscle triacylglycerol content and (**d**) glycogen content (n=10). See ESM Fig. 6 for full-length western blots. Kruskal-Wallis test was performed for (**a–d**). Data are represented as mean ± SD. Bars indicate respective post hoc analysis.

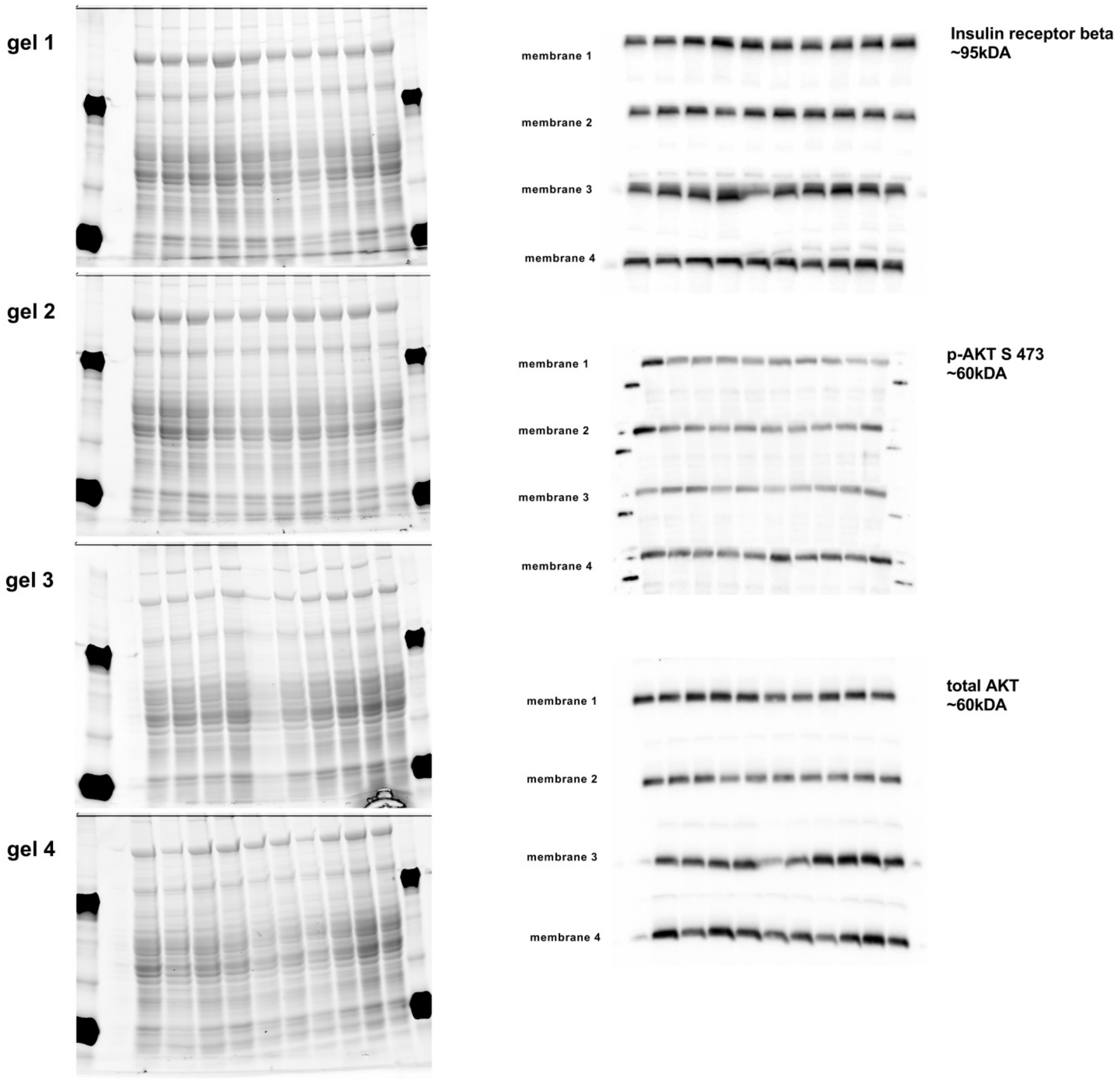


**ESM Fig. 4. Full-length western blots of Fig. 4.** On the left side loading controls are depicted for each corresponding membrane using the stain free technology.

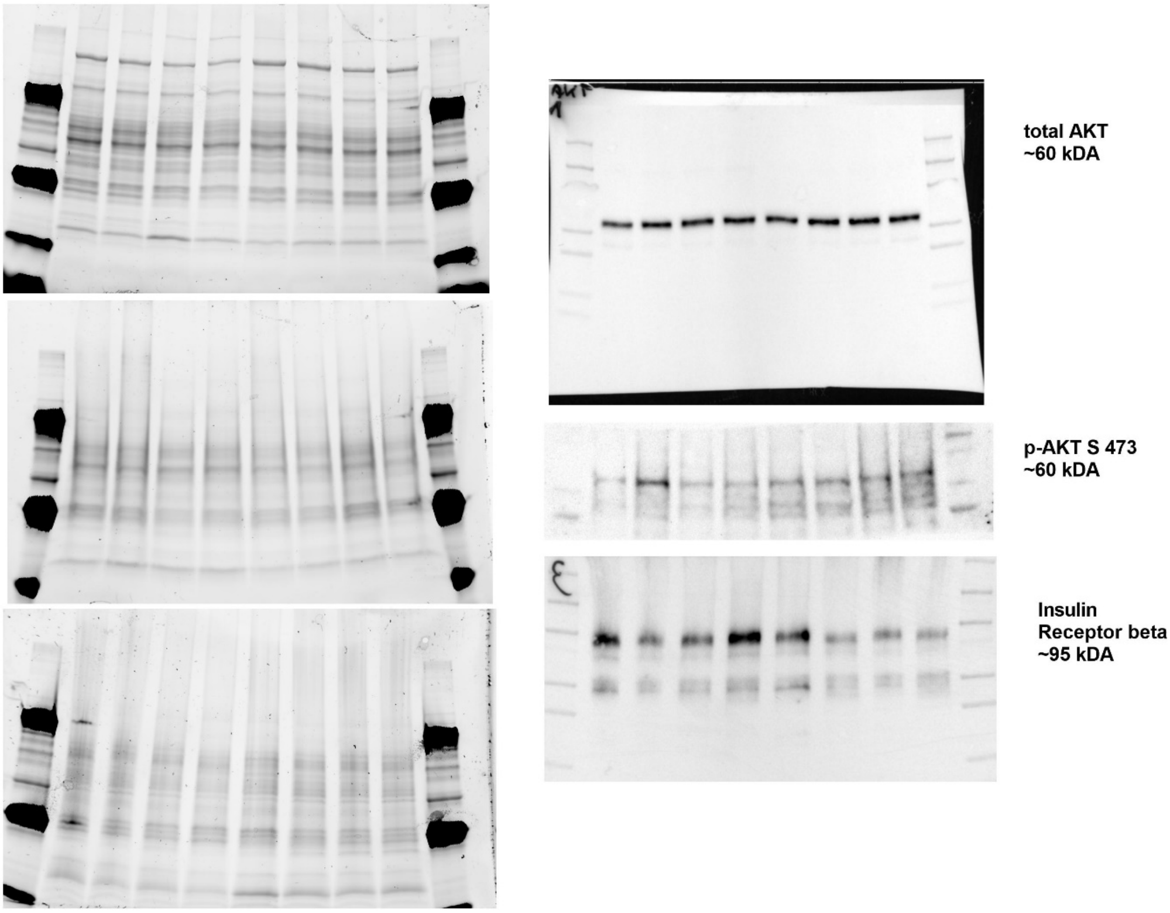


**ESM Fig. 5. Full-length western blots of Fig. 4 (Representative Images).** On the left side loading controls are depicted for each corresponding membrane using the stain free technology

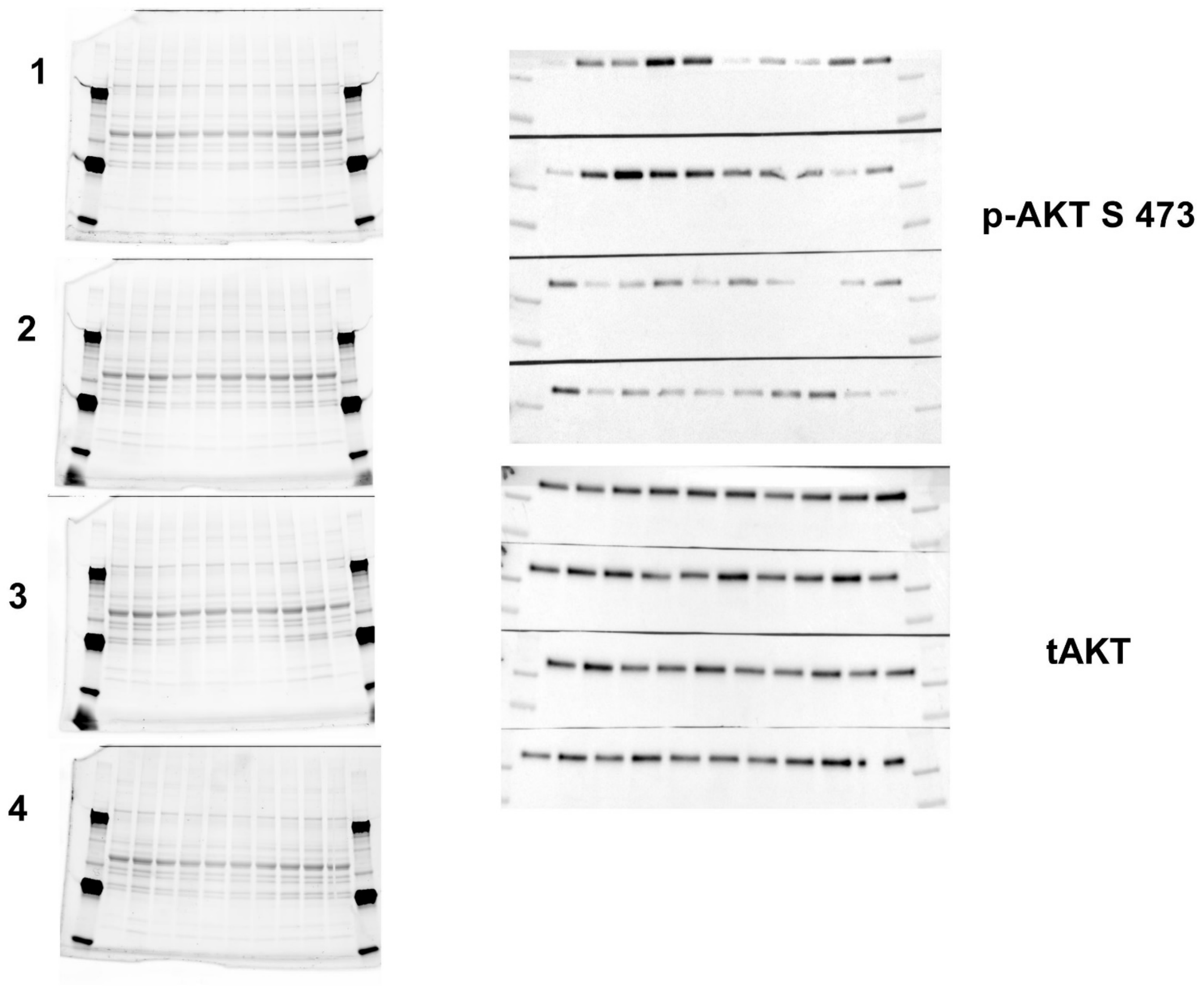


**ESM Fig. 6. Full-length western blots of ESM Fig. 3.** On the left side loading controls are depicted for each corresponding membrane using the stain free technology
